# Proinflammatory cytokine profiles in prediabetic Saudi patients

**DOI:** 10.1016/j.sjbs.2023.103714

**Published:** 2023-06-26

**Authors:** Nouf O. AlAfaleq, Tasneem M. Hussein, Samia T. Al-Shouli, Nojood Altwaijry, Mohd Shahnawaz Khan, Aqel Albutti, Maaweya E. Hamed

**Affiliations:** a Department of Biochemistry, College of Science, King Saud University, Riyadh, Saudi Arabia; b Immunology Unit, Department of Pathology, College of Medicine, King Saud University, Riyadh, Saudi Arabia; c Department of Medical Biotechnology, College of Applied Medical Sciences, Qassim University, Buraydah, Saudi Arabia; d Research Center, King Fahad Medical City, Riyadh, Saudi Arabia

**Keywords:** Prediabetes, Inflammation, Cytokines, Gene expression profiling, Saudi patients

## Abstract

Prediabetes is an increase-risk state for diabetes that is associated with an increase in blood glucose levels to more than normal, but not increased enough to be termed as type 2 diabetes mellitus (T2DM). A timely intervention and management of prediabetes can stop its further progression to the diabetic state. Many cytokines are involved in diseases including diabetes, however, their role in prediabetes is unknown. In this study, we attempted to analyze numerous proinflammatory cytokines in prediabetic patients. A total of 60 adult Saudi prediabetes patients and healthy control individuals were included in this study. To better understand the role of the proinflammatory cytokines in prediabetes patients and its potential link to the disease outcome, the variations in the levels of these cytokines were investigated using Multi-Analyte ELISA technique. The T helper cells (Th1 and Th2) immune response expression profiling of 84 genes was done using Real Time-quantitative PCR (RT-qPCR) technique. The present finding showed that serum Interleukin IL-2, IL-1β, and IL-1α levels of all prediabetes patients were increased when compared with healthy control cases (P < 0.05). Inductions of proinflammatory cytokines and upregulation of Th1 and Th2 immune genes might play a potential role during prediabetes status and may be linked to the disease outcome. Further studies are needed to investigate the underlying mechanism of these proinflammatory cytokines in diabetes development. A strong positive correlation was found between IL and 1α with glucose levels than with IL-1β and IL-2. In conclusion, cytokines, especially IL-1, may play a critical role in the development of diabetes.

## 1 Introduction

The potential of the immune system to differentiate between non-self and self relies on the intricate regulation of cellular responses to T helper cells (Th) specifically Th1 and Th2 populations (Pearson and McDevitt, 1999). Th1 cells are involved in activation of macrophages to a microbicidal phase and the enhancement of the cellular immunity against virus or intracellular pathogens (Sacks and Noben-Trauth, 2002). While Th2 cells are important for the stimulation and development of immune functions (Pulendran and Artis, 2012). The balance between Th1 and Th2 is affected in several immunological disorders, favoring Th1 or Th2 response. The comparison of Th1 and Th2 responses in the context of autoimmune disease shows that the relative contribution of either T cell type to the formation of a specific autoimmune response can affect the outcome (Ruterbusch et al., 2020). Hence, methods that control the relative contributions of Th1 and Th2 cells to an autoimmune response may be used to control autoimmune disease.

Diabetes is a prevalent autoimmune condition affecting millions of people globally (Boles et al., 2017). The inflammatory response persists in many chronic diseases including diabetes and causes severe tissue and organ damage (Duan et al., 2019). It is very important to consider the traits of various T cell subsets, the balance between them, as well as their flexibility in order to comprehend the involvement of proinflammatory cytokines in autoimmunity (Mosmann and Sad, 1996).

The important proinflammatory cytokines are IL-1, IL-6, IL-18, and TNF-α. Proinflammatory cytokines were found to be overexpressed in numerous autoimmune and systemic inflammatory disorders (Gulati et al., 2016). An improved understanding of a disease's pathogenesis may result from measuring proinflammatory cytokines (Turner et al., 2014). Several studies have shown that the balance between the Th1 and Th2 immune response has a significant impact on disease progress (Dinarello, 2009). The IL-1 family is more than any other cytokine family associated with innate immunity (Dinarello, 2018). Many studies have revealed that the presence of adhesion molecules is increased in the glomerular endothelium and that these molecules are expressed in other kidney structures by IL-1 (Duran-Salgado and Rubio-Guerra, 2014). In addition, IL-1 promotes the synthesis of hyaluronan, which results in cell proliferation in diabetics and supports the onset of diabetic nephropathy. This proinflammatory cytokine is known to be elevated in experimental models of albuminuria and concurrent macrophage buildup (Leehey, 2020). Reactive oxygen species (ROS) and impaired function of mitochondria are involved in the development of diabetes. The increased amounts of ROS and induced oxidative stress mediate the complications of diabetes (Navarro-González and Mora-Fernández, 2008). For example, pancreatic islets have been found to secrete IL-1 and cytokine levels are markedly elevated in the inflammatory state in type 2 diabetes or autoimmune type 1 diabetes (Weksler-Zangen et al., 2008).

By 2030, more than 470 million individuals will have prediabetes, as the prevalence of prediabetes continues to rise globally. About 25% of people with prediabetes may eventually develop type 2 diabetes within 3–5 years (Cai et al., 2020). Lifestyle is one of the major factors that influence the increase of diabetic cases in recent years. There is an assumption that medical intervention can prevent the progression to diabetes by identifying people with prediabetes. The objective of the present study was to analyze inflammatory cytokines in prediabetic patients.

## 2 Materials and methods

### 2.1 Subjects and sample collection

Samples of 30 Saudi prediabetes patients, ages 18–60 years old, were collected from King Saud University Hospital. Healthy participants matching in age and gender, were used in the study as controls. Participants with chronic inflammatory conditions were excluded. Patients with fasting glucose levels in the range of 100–125 mg/dl were considered prediabetic. Patients with A1C (hemoglobin A1C) levels in the range of 5.7% to 6.4% were considered prediabetic. A1C was used to predict blood glucose levels of the cases for the past 90 days. Blood samples (5 mL) from fasting individuals were collected in tubes containing EDTA. The samples were then stored at 2 – 8 °C until they were processed. The collected blood was centrifuged at 5000 rpm for 10 min and plasma was obtained. It was stored at −80 °C for further study. Plasma was used for the analysis by enzyme-linked immunosorbent assay (ELISA). Peripheral blood mononuclear cells (PBMC) were used for RNA extraction and quantitative RT^2^ profiler™ PCR Array polymerase chain reaction.

### 2.2 Isolation of mononuclear cells

Ficoll (or Ficoll-Paque) was used to isolate PBMC (Panda and Ravindran, 2013). Briefly, a total of 2 mL Ficoll was mixed with blood samples and centrifuged at 5000 rpm for 10 min at 4 °C. A total of four layers developed and the top layer containing plasma, was gently removed, and stored. PBMCs, typically white, hazy “blanket,” constitute the second layer. Using a Pasteur pipette, these cells were carefully taken out and stored at −80 °C for isolation of RNA.

### 2.3 Total RNA purification and reverse-transcription PCR (RT-PCR)

PBMC cells were used to extract total RNA using a commercial RNA kit (RNeasy Mini kit) (Qiagen, Germany). The TaqMan Reverse Transcription Reagents and RT Reaction Mix were used to reverse-transcribe RNA. Genomic DNA contamination was removed by DNase treatment. RNA was measured and its integrity was evaluated using a Nano Drop spectrophotometer (Thermo Scientific, Waltham, USA).

### 2.4 RT^2^ profiler PCR arrays

Measurement of the pulmonary Th1 and Th2 immune response mRNA gene expression. The RT^2^ First Strand Kit was used to perform reverse transcription on whole mRNA (SA-Biosciences, Hilden, Germany). First-strand cDNA was employed for RT^2^ profiler Real Time PCR. Using the RT^2^ profiler TM PCR Array Gene Expression Assay (Qiagen, Cat#, PAHS-034) and the RT^2^ SYBR Green qPCR master mix in accordance with the manufacturer's protocols. The genes expression associated with T helper (adaptive) immune responses was assessed. To determine the expression changes (fold) for each gene in comparison to the housekeeping genes, the Ct technique was used. The result was processed utilizing SA Biosciences software for RT^2^ Profiler PCR Array data analysis. The average difference in gene expression between the control and each of the 84 genes was used to express fold changes. Two-fold or more difference was considered statistically significant.

### 2.5 Real-time PCR array

Five distinct housekeeping genes (HSP90AB1, HPRT, GAPDH, GUSB, and ACTB) were present on each array and were utilised to normalise the sample data. Calculating the variation between the cycle thresholds for the specific gene and the genes used for normalization (housekeeping gene) (Ct) were allowed for normalisation to the housekeeping genes (HKG). The ideal reference genes for normalising the PCR arrays gene expression data were determined using the RT^2^ Profiler PCR Array data processing programme. The RT^2^ PCR Profiler Data PCR array analysis software produced a heat map, scatter-plots, chart, and line graph.

### 2.6 Multiplex immunoassay of inflammatory mediators

The Multi-Analyte ELISArray Kit was used to analyze the presence of 12 human cytokines associated with inflammation in plasma samples from prediabetes patients and healthy controls (Qiagen). The manufacturer's instructions were followed for measuring the primary 12 human proinflammatory cytokines from both prediabetes patients and the healthy non-diabetes control group. After 30 min incubation, the resulting products were measured at 450 nm, and the presence of cytokines was determined by comparing them to the positive and negative controls. Based on an absorbance value above the negative control, the presence of cytokine was determined meanwhile the average absorbance of all standards and samples was determined using replicates.

### 2.7 Statistical analysis

For the statistical analysis of the ELISA Array results, GraphPad prism software was used to determine unpaired T test. P-value < 0.05 was considered significant.

## 3 Results

### 3.1 Prediabetes is associated with proinflammatory cytokine induction

**Fig. 1:**
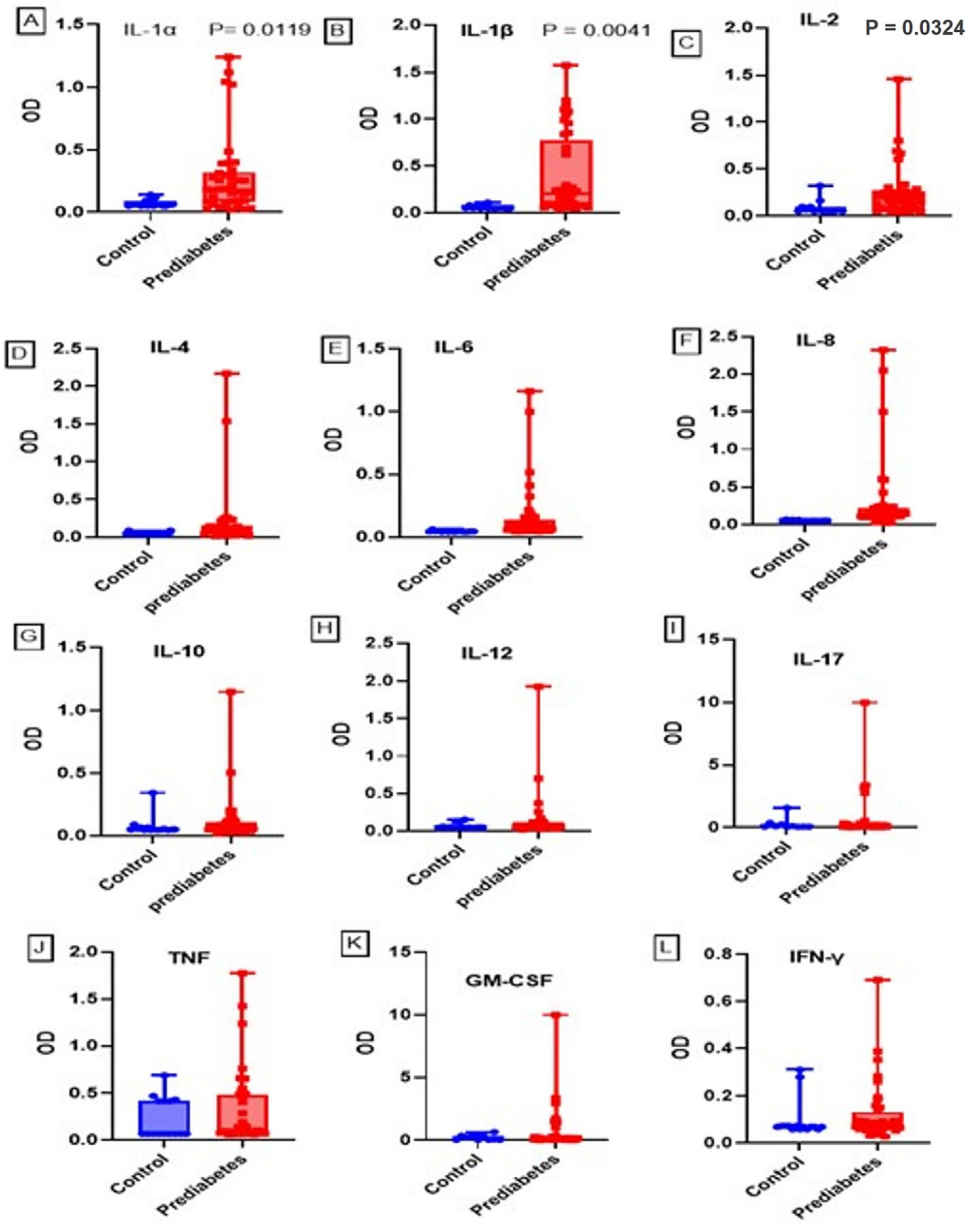
Human proinflammatory cytokines were measured by multiplex ELISA array in prediabetes and healthy control group (A) IL-1α, (B) IL-1β, (C) IL-2, (D) IL-4, (E) IL-6, (F) IL-8, (G) IL-10, (H) IL-12, (I) IL-17 (J) TNF, (K) GM-CSF and (L) IFN-γ. The results are expressed as optical density.

In prediabetes cases levels of cytokines: IL-2, IL-1β, and IL-1α were observed to be significantly higher than control group (Fig. 1). No significant variation of cytokines was observed between prediabetes and control group for IL-6, IL-10, IL-17A, IL-4, IL-8, IFN-γ, and TNF-α. The results are expressed as optical density (OD).

### 3.2 Th1 and Th2 cytokines genes were upregulated during prediabetic stage

**Fig. 2:**
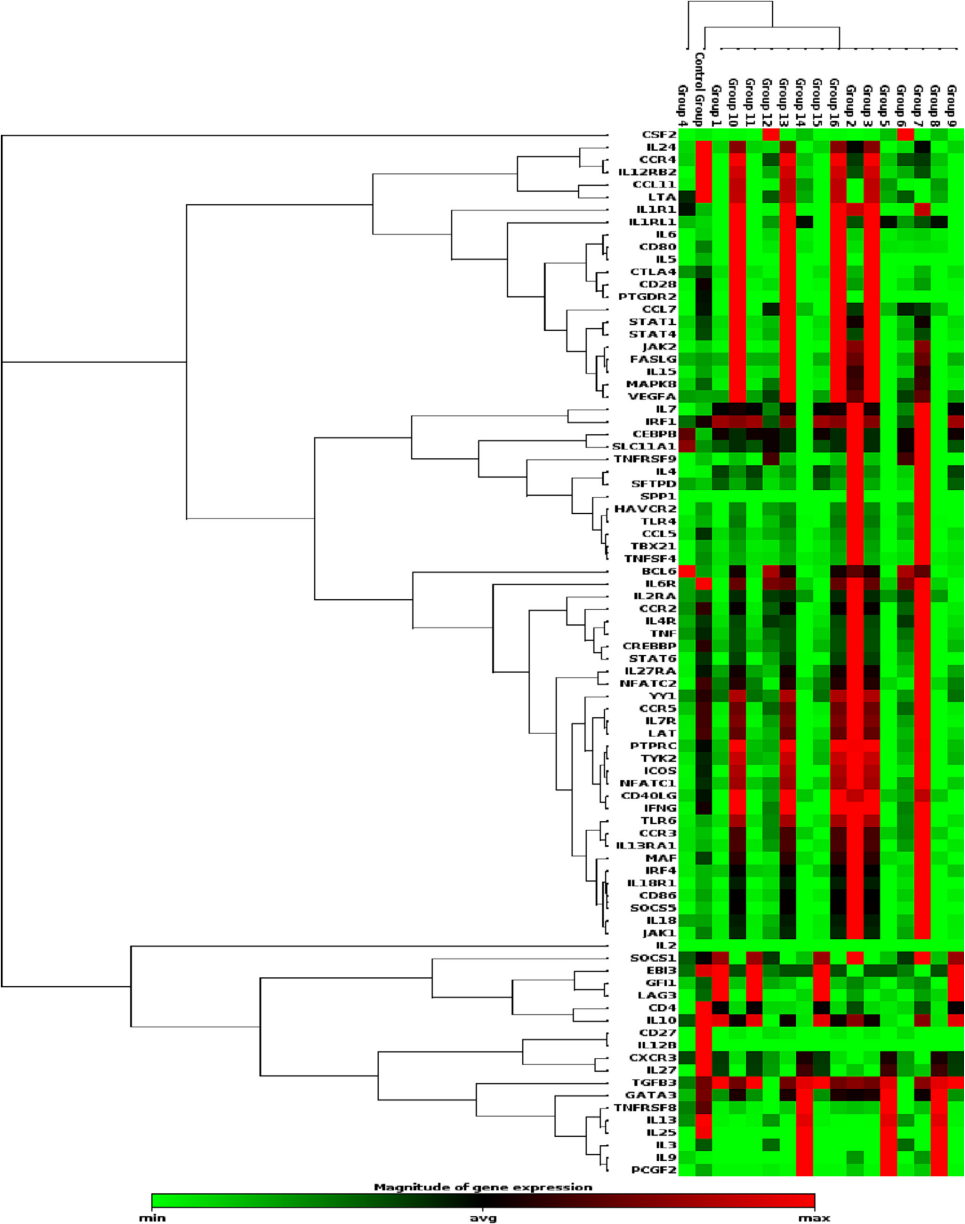
A Heat map providing a graphical representation of gene expression change in the prediabetes group against the control group. Gene upregulation and down regulation are represented by the red and green colours, respectively. The variation of colour (green to red) shows variations of genes expressions. Data was analyzed by RT^2^PCR Profiler Data PCR array software.

To study the gene expression of cytokines, RT^2^-PCR Array was used. An upregulation was found in BCL6 (2 fold), CCR3 (2.6-fold), CSF2 (3.8 fold), IL-1R1 (2 fold), IL-4 (5 fold), IL-5 (1.3 fold), IL-6 (2 fold), IL-9 (2.6 fold), Janus kinase 2 (JAK2) (3 fold), and VEGFA (2 fold). A downregulation of<2 fold in expression was noted for IL-15 (1.7 fold), IL-18 (1.4 fold), SOCS5 (1.8 fold), TLR4 (1.3 fold), TLR6 (1.7 fold) and TNFRSF9 (1.6 fold) (Fig. 2).

## 4 Discussion

There is an assumption that intervention can stop the progression of diabetes by identifying people with prediabetes. Many cytokines were reported in numerous inflammatory and non-inflammatory disease conditions including diabetes, however, their role in the prediabetes state is unknown, hence the need for investigation. In this study, increased proinflammatory cytokines were detected in prediabetes patients. In many studies, the levels of these cytokines have been shown to be highly expressed in diabetes mellitus patients, suggesting a role in diabetes progression. These cytokines have also been related to diabetes complications reported by previous studies (Downes et al., 2014). Our findings showed that IL-1α, IL-1β and IL-2 were significantly elevated in individuals with prediabetes when compared with normal controls (Fig. 1). In accordance, IL-1α has been used as a predictor biomarker for several chronic and autoimmune diseases such as sepsis, diabetes, and nephropathy (Sindhughosa and Pranamartha, 2017). In this study high expression of certain genes were shown, which has also been reported in diabetic retinopathy patients (Takeuchi et al., 2015). In diabetes, an elevated level of IL-18 was reported, and this cytokine is also used to predict the risk of cardiovascular diseases. In addition, the variation of IL-18 in the blood is associated with insulin resistance and the level of IL-18 in the bloodstream is associated with the physical exercise of the individual. In this experiment the upregulation of IL‐18 has been shown, which is in line with several studies showing IL-18 elevation and upregulation in diabetes patients (Yaribeygi et al., 2018) (Miyauchi et al., 2009).

Results of this study and previous results showed an overexpression of Toll-like receptors (TLR4 and 6), Janus kinase 2 (JAK2) and Vascular endothelial growth factor A (VEGFA) in prediabetes patients (Guo et al., 2018). It has been demonstrated that the STAT-dependent IL-4 responses of B cells are modulated by the B-Cell Lymphoma 6 (BCL6) protein, which functions as a sequence-specific transcriptional repressor (Arima et al., 2008). In this study the mRNA expression of BCL6 was found to be upregulated in patients associated with prediabetes disease. CSF2 plays a role in granulocyte and macrophage activation

CCR3 is responsible for recruitment of inflammatory cells such as eosinophils, Th2 cell subsets, basophils, and mast cells (Romagnani, 2002). In our study, the increased expression of CCR3, CSF2 and SOCS5 mRNA was observed. Previous studies have reported that CCR3 was upregulated in diabetes patients (Alblowi et al., 2013). The present results are consistent with previous published results and provide additional proinflammatory cytokines profiling.

## 5 Conclusion

The results showed that the prediabetes stage could be associated with elevated levels of tested cytokines in plasma since 10 out of 84 Th1/2 related genes in PBMCs were significantly increased in prediabetes than in healthy individuals. Proinflammatory cytokines and the upregulation of gene expression Th2 and Th1 immune responses in the prediabetes stage, could be a major output in the diabetes research. Further studies should be done to follow up the prediabetes patients and to determine the impact of Th1 and Th2 immune responses and various cytokines in diabetes development.

## 6 Institutional board statement

This project was approved by Institutional Ethical Committee, King Saud University College of Medicine (IRB register number 19–4256).

## 7 Informed consent statement

Informed consent was obtained from all individual participants included in the study.

## Declaration of Competing Interest

The authors declare that they have no known competing financial interests or personal relationships that could have appeared to influence the work reported in this paper.

## References

[b0005] Alblowi J., Tian C., Siqueira M.F., Kayal R.A., McKenzie E., Behl Y., Gerstenfeld L., Einhorn T.A., Graves D.T. (2013). Chemokine expression is upregulated in chondrocytes in diabetic fracture healing. Bone.

[b0010] Arima M., Fukuda T., Tokuhisa T. (2008). Role of the transcriptional repressor BCL6 in allergic response and inflammation. World Allergy Organ. J..

[b0015] Boles A., Kandimalla R., Reddy P.H. (2017). Dynamics of diabetes and obesity: Epidemiological perspective. Biochim. Biophys. Acta Mol. Basis Dis..

[b0020] Dinarello C.A. (2009). Immunological and inflammatory functions of the interleukin-1 family. Annu. Rev. Immunol..

[b0025] Dinarello C.A. (2018). Overview of the IL-1 family in innate inflammation and acquired immunity. Immunol. Rev..

[b0030] Downes K., Marcovecchio M.L., Clarke P., Cooper J.D., Ferreira R.C., Howson J.M., Jolley J., Nutland S., Stevens H.E., Walker N.M., Wallace C., Dunger D.B., Todd J.A. (2014). Plasma concentrations of soluble IL-2 receptor α (CD25) are increased in type 1 diabetes and associated with reduced C-peptide levels in young patients. Diabetologia.

[b0035] Duan L., Rao X., Sigdel K.R. (2019). Regulation of inflammation in autoimmune disease. J. Immunol. Res..

[b0040] Duran-Salgado M.B., Rubio-Guerra A.F. (2014). Diabetic nephropathy and inflammation. World J. Diabetes.

[b0045] Gulati K., Guhathakurta S., Joshi J.C., Rai N., Ray A. (2016). Cytokines and their role in health and disease: A brief overview. MOJ Immunol..

[b0050] Guo B.Q., Xu J.B., Xiao M., Ding M., Duan L.J. (2018). Puerarin reduces ischemia/reperfusion-induced myocardial injury in diabetic rats via upregulation of vascular endothelial growth factor A/angiotensin-1 and suppression of apoptosis. Mol. Med. Rep..

[b0055] Leehey D.J. (2020). Targeting inflammation in diabetic kidney disease: Is there a role for pentoxifylline?. Kidney360.

[b0060] Miyauchi K., Takiyama Y., Honjyo J., Tateno M., Haneda M. (2009). Upregulated IL-18 expression in type 2 diabetic subjects with nephropathy: TGF-beta1 enhanced IL-18 expression in human renal proximal tubular epithelial cells. Diabetes Res. Clin. Pract..

[b0065] Mosmann T.R., Sad S. (1996). The expanding universe of T-cell subsets: Th1, Th2 and more. Immunol. Today.

[b0070] Panda S.K., Ravindran B. (2013). Isolation of human PBMCs. Bio-protocol.

[b0075] Pearson C.I., McDevitt H.O. (1999). Redirecting Th1 and Th2 responses in autoimmune disease. Curr. Top. Microbiol. Immunol..

[b0080] Pulendran B., Artis D. (2012). New paradigms in type 2 immunity. Science.

[b0085] Romagnani S. (2002). Cytokines and chemoattractants in allergic inflammation. Mol. Immunol..

[b0090] Ruterbusch M., Pruner K.B., Shehata L., Pepper M. (2020). In vivo CD4(+) T cell differentiation and function: Revisiting the Th1/Th2 paradigm. Annu. Rev. Immunol..

[b0095] Sacks D., Noben-Trauth N. (2002). The immunology of susceptibility and resistance to Leishmania major in mice. Nat. Rev. Immunol..

[b0100] Sindhughosa D.A., Pranamartha A.A.G.M.K. (2017). The involvement of proinflammatory cytokines in diabetic nephropathy: Focus on interleukin 1 (IL-1), interleukin 6 (IL-6), and tumor necrosis factor-alpha (TNF-α) signaling mechanism. Bali Med. J..

[b0105] Takeuchi M., Sato T., Tanaka A., Muraoka T., Taguchi M., Sakurai Y., Karasawa Y., Ito M. (2015). Elevated levels of cytokines associated with Th2 and Th17 cells in vitreous fluid of proliferative diabetic retinopathy patients. PLoS One.

[b0110] Turner M.D., Nedjai B., Hurst T., Pennington D.J. (2014). Cytokines and chemokines: At the crossroads of cell signalling and inflammatory disease. Biochim. Biophys. Acta.

[b0115] Yaribeygi H., Mohammadi M.T., Rezaee R., Sahebkar A. (2018). Crocin improves renal function by declining Nox-4, IL-18, and p53 expression levels in an experimental model of diabetic nephropathy. J. Cell. Biochem..

